# Clinical usefulness and validity of robotic measures of reaching movement in hemiparetic stroke patients

**DOI:** 10.1186/s12984-015-0059-8

**Published:** 2015-08-12

**Authors:** Eri Otaka, Yohei Otaka, Shoko Kasuga, Atsuko Nishimoto, Kotaro Yamazaki, Michiyuki Kawakami, Junichi Ushiba, Meigen Liu

**Affiliations:** Department of Rehabilitation Medicine, Keio University School of Medicine, Tokyo, Japan; Department of Biosciences and Informatics, Faculty of Science and Technology, Keio University, Kanagawa, Japan

## Abstract

**Background:**

Various robotic technologies have been developed recently for objective and quantitative assessment of movement. Among them, robotic measures derived from a reaching task in the KINARM Exoskeleton device are characterized by their potential to reveal underlying motor control in reaching movements. The aim of this study was to examine the clinical usefulness and validity of these robot-derived measures in hemiparetic stroke patients.

**Methods:**

Fifty-six participants with a hemiparetic arm due to chronic stroke were enrolled. The robotic assessment was performed using the Visually Guided Reaching (VGR) task in the KINARM Exoskeleton, which allows free arm movements in the horizontal plane. Twelve parameters were derived based on motor control theory. The following clinical assessments were also administered: the proximal upper limb section in the Fugl-Meyer Assessment (FMA-UE(A)), the proximal upper limb part in the Stroke Impairment Assessment Set (SIAS-KM), the Modified Ashworth Scale for the affected elbow flexor muscles (MAS elbow), and seven proximal upper limb tasks in the Wolf Motor Function Test (WMFT). To explore which robotic measures represent deficits of motor control in the affected arm, the VGR parameters in the paretic arm were compared with those in the non-paretic arm using the Wilcoxon signed rank test. Then, to explore which VGR parameters were related to overall motor control regardless of the paresis, correlations between the paretic and non-paretic arms were examined. Finally, to investigate the relationships between the robotic measures and the clinical scales, correlations between the VGR parameters and clinical scales were investigated. Spearman’s rank correlation coefficients were used for all correlational analyses.

**Results:**

Eleven VGR parameters on the paretic side were significantly different from those on the non-paretic side with large effect sizes (|effect size| = 0.76–0.87). Ten VGR parameters correlated significantly with FMA-UE(A) (|r| = 0.32–0.60). Eight VGR parameters also showed significant correlations with SIAS-KM (|r| = 0.42–0.49), MAS elbow (|r| = 0.44–0.48), and the Functional Ability Scale of the WMFT (|r| = 0.52–0.64).

**Conclusions:**

The robot-derived measures could successfully differentiate between the paretic arm and the non-paretic arm and were valid in comparison to the well-established clinical scales.

## Background

Reaching is an essential function for many activities of daily living (ADL) such as feeding, toileting, grooming, and dressing, but is commonly impaired in post-stroke patients. It is therefore necessary for rehabilitation practitioners to understand the details of normal and impaired reaching movement. Reaching requires not only multiple-joint coordination but also involvement of the central nervous system (CNS) to plan strategies to control the musculoskeletal system. Because of its complexity and redundancy, reaching movement is difficult to quantify in clinical practice [1]. In clinical settings, motor impairment after a stroke is usually assessed with clinical scales specific to CNS lesions such as the Brunnstrom Recovery Stages [2], the Fugl-Meyer Assessment (FMA) [3], and the Chedoke-McMaster Stroke Assessment (CMSA) [4], which were developed based on the typical motor recovery patterns of hemiparesis. On the other hand, the function of the upper extremities can be assessed with various task-based instruments such as the Action Research Arm Test [5], the Box Block Test [6], and the Wolf Motor Function Test (WMFT) [7]. Those tests score function on a ‘can’ or ‘cannot’ basis or in terms of the time required to complete a task. However, the relationship between the reaching function itself and those well- established assessments for impairment and/or function is not fully understood. Furthermore, these clinically used assessments cannot provide any information about the underlying strategies and mechanisms of impaired motor control in stroke patients.

Recently, various kinds of robotic technologies have been developed for more accurate assessment of reaching movement. Generally, robotic assessments can be objective, quantitative, and continuous scales with less floor or ceiling effects compared to conventional clinical tools [8]. According to Balasubramanian et al. [9], currently existing movement measures used in rehabilitation robotics are classified into three broad categories: kinematic measures (e.g., movement deviation, time, and velocity), kinetic measures (e.g., force direction error and amount of assistance), and neuromechanical measures (e.g., arm impedance). Several robots such as the RUPERT (Robotic assisted UPper Extremity Repetitive Therapy) device [10], MEMOS (MEchatronic system for MOtor recovery after Stroke) [11], and the InMotion2 robot (Interactive Motion Technologies, Inc.) [12]—which is a commercial version of the MIT-Manus [13]—can provide multiple kinematic and kinetic measures from a single task. Those measures, however, are not enough to assess the whole motor control system involved in reaching movements. On the other hand, Coderre et al. [14] present another classification of robotic measures based on motor control theory: postural control, visual reaction, feed-forward control (initiation of movement), feedback control (corrective responses), and total movement metrics. This approach may be able to reveal directly the underlying strategies of the motor control system in reaching movements. We can obtain these robotic measures from a task called Visually Guided Reaching (VGR) among the standard tasks of the KINARM Exoskeleton (BKIN Technologies Ltd, Kingston, ON, Canada) [14, 15].

Although these new robot-derived measures can potentially add a new dimension to existing rehabilitation practice, they need to be sufficiently validated and revealed to have clinical usefulness and importance before being used in practice. As for the KINARM Exoskeleton, the validity of discriminability between normal and abnormal in stroke patients has been reported [14, 16]. However, the correlations between robotic measures and clinical scales assessing impairments in the paretic arm have yet to be examined. For clinical use, it is very important to ascertain that the robotic measures can successfully vary in accordance with the severity of the paresis and to know what indices among various robotic measures are well correlated with the degree of paresis. It has also been reported that the VGR parameters were correlated with the Purdue Pegboard (PPB) and the Functional Independence Measure (FIM) [16]. However, validation of the VGR parameters with clinical assessments focused on arm function has not been well examined because the PPB was developed for the evaluation of manipulative dexterity rather than reaching [17]. Moreover, FIM, an evaluation of ADLs, is influenced by various factors such as the ability of the trunk and of the unaffected side [18] as well as the severity of impairment in the affected arm. Therefore, the VGR parameters derived from the KINARM Exoskeleton remain to be validated with well-established clinical assessments mainly focused on arm impairment and/or function.

In addition, one of the characteristics of this KINARM Exoskeleton is that it provides full gravitational support and permits arm movement only in the horizontal plane with reduced degrees of freedom: only one degree of freedom each in the shoulder and the elbow. Therefore, tasks in the KINARM Exoskeleton might be easier to perform than real, three-dimension tasks under the gravity for the patients with hemiparesis. This is an advantage of this robotic device in that even severe hemiparetic patients can be assessed. Furthermore, reduced degree of freedom may reduce movement redundancy resulting in less compensatory movements. Hence the movement on this robotic device may reflect true impairments and may be robust indices for the impairments. However, here raises the questions about whether these parameters obtained from the task performed in reduced degree of freedom with gravitational supports can provide any useful information about the real movement performed in the three-dimension.

The purpose of this study is to examine the clinical usefulness and the validity of the reaching task in KINARM Exoskeleton in hemiparetic stroke patients. More specifically, the aims of the study are (1) to explore which robotic measures of reaching movements represent the deficits of motor control in the arm and which measures are related to the overall motor control regardless of the paresis, and (2) to examine the correlation between robotic measures and well-established clinical assessments of impairment and function.

## Methods

### Participants

Patients with a hemiparetic arm due to chronic stroke were recruited among those hospitalized for HANDS (hybrid assistive neuromuscular dynamic stimulation) therapy [19] at the Department of Rehabilitation Medicine at Keio University Hospital during the period from November 2012 to January 2015. Inclusion criteria were as follows: more than 150 days since stroke onset, no clinically obvious cognitive deficits, living independently, no pain in the paretic upper extremities, ability to maintain the sitting position with no difficulty, and sufficient range of motion to perform tasks in KINARM. Patients were excluded from the study if exercise of the upper limbs was prohibited for medical reasons or if the paretic hand could not be raised to the height of the nipples in the sitting position because of severe hemiparesis. Patients with foreign materials (pacemakers, shunts, or clipping) or a history of epilepsy were also excluded. The study protocol was approved by the institutional ethics committee (#20120070) and registered at the UMIN Clinical Trial Registry (UMIN000009269). All participants gave informed consent according to the Declaration of Helsinki. Characteristics of the participants are shown in Table 1.

**Table 1:** Characteristics of the participants

**Characteristic**		**(N = 56)**
Age, mean ± standard deviation (range)		49.4 ± 11.8 (18–78)
Sex, male/female		37/19
Type of disease	Cerebral infarction	22
	Cerebral hemorrhage	32
	Subarachnoid hemorrhage	2
Days after the onset of hemiparesis, median (range)		548.5 (164–6456)
FMA-UE (A), median (range)		22 (5–35)
SIAS-KM, median (range)		3 (2–4)
MAS elbow, median (range)		1 (0–2)
		[10, 19, 24, 3, 0, 0]^a^
WMFT_FAS, median (range)		35 (22–58)

*FMA-UE(A)* Part A of the Fugl-Meyer Assessment Upper Extremity, *SIAS-KM* Knee-Mouth test of the Stroke Impairment Assessment Set *MAS* Modified Ashworth Scale, *WMFT-FAS* Functional Ability Scale of the Wolf Motor Function Test. ^a^The number of participants in each category of the MAS [0, 1, 1+, 2, 3, 4]

### Robotic assessment of the reaching task

The robotic assessment was performed using the KINARM Exoskeleton. The device has one degree of freedom for each joint (shoulder and elbow): elbow flexion/extension and horizontal shoulder adduction/abduction. This device permits free arm movement in the horizontal plane involving flexion and extension of the shoulder and elbow by providing weight support to the arms on the metallic framework with plastic troughs. Visual feedback can be given on the virtual reality display just above the plane of the arms. Participants were initially seated in the KINARM Exoskeleton with their shoulders abducted. Then the experimenter adjusted the exoskeleton, which consisted of plastic arm troughs for each arm segment (arm, forearm, and hand), so that it fit comfortably and the arms were in the same plane as the shoulder (80–90° abducted).

Participants performed the VGR (a reaching task), one of the standard tasks in the KINARM Exoskeleton. The goal of the task was to make unassisted reaching movements quickly and accurately from a centrally located target (1.0-cm radius) to one of eight peripheral targets (1.0-cm radius) distributed uniformly on the circumference of a circle (with 10 cm from the center target to each peripheral target) by moving the shoulder and the elbow. The position of the tip of the index finger was tracked using a computerized representation (a small white circle, 0.4 cm radius) as visual feedback. To characterize performance, twelve movement parameters based on motor control theory were calculated from each trial. Details of the task and parameters used in this study have been described by Coderre et al. [14]. The parameters are presented in Table 2.

**Table 2:** The parameters of the visually guided reaching task

Parameters	Description
Upper-limb postural control	
Posture Speed (m/s)	The mean hand speed for 500 ms before peripheral target illumination, when the hand should be at rest.
Visual reaction	
Reaction Time (s)	The time between illumination of the peripheral target and the onset of movement.
No Reaction Time	The number of trials for which movement to the destination target could not be detected.
Feed-forward control (initiation of movement)	
Initial Direction Error (radian)	The angular deviation between (a) a straight line from the hand position at movement onset to the destination target and (b) a vector from the hand position at movement onset to the hand position after the initial phase of movement.
Initial Distance Ratio	The ratio of (a) the distance the hand traveled during the participants’ initial phase of movement to (b) the distance the hand traveled between movement onset and offset.
Initial Speed Ratio	The ratio of (a) the maximum hand speed during the participant’s initial phase of movement to (b) the maximum hand speed during the trial.
Feed-back control (corrective responses)	
Speed Maxima Count	The number of maxima in hand speed velocity between movement onset and offset.
Min-Max Speed (m/s)	The differences between local speed peaks and minima.
Total movement metrics	
Movement Time (s)	The total time elapsed from movement onset to offset.
Path Length Ratio	The ratio of (a) the distance travelled by the hand between the movement onset and offset and (b) the straight-line distance between the starting and destination targets.
Max Speed (m/s)	The maximum hand speed during the trial.
No Movement End	The number of trials for which a stopping on the destination target was not detected.

“Initial phase of movement” denotes the time from movement onset to the time of the first speed minimum

“Movement offset” indicates the time at which the participant finished their movement to the destination target; if the destination target was not reached, then movement offset is not calculated

### Clinical assessments

Participants were examined with the following clinical instruments:

1. Fugl-Meyer Assessment (FMA) [3]

This is one of the most widely used quantitative measures of motor impairment in stroke patients with high inter-rater reliability [20, 21] and validity [22]. This measure offers five domains (motor function, sensory function, balance, joint range of motion, joint pain) to assess synergistic and voluntary movement after stroke in about thirty minutes. A three-point ordinary scale is used to assess movement (0 = unable; 1 = partial; 2 = performs fully) in each item. In this study, we only used part A of the upper limb section (shoulder/elbow/forearm) in the motor function domain (FMA-UE(A)) for the analyses. Since the FMA-UE(A) consists of eighteen test items, the score ranges from 0 (worst) to 36 (best).

2. Stroke Impairment Assessment Set (SIAS) [23]

This evaluation tool was developed and is widely used in Japan to assess various aspects of impairment in hemiplegic patients, including motor function, tone, sensory function, range of motion, pain, trunk function, visuospatial function, speech, and sound side function [23]. Its reliability, validity, and responsiveness have been reported previously [23, 24]. In this study, we used part of it for assessing motor function of proximal upper extremities: the Knee-Mouth Test (SIAS-KM). In the sitting position, the participant repeated lifting the affected hand from the contralateral knee to the mouth so that the affected-side shoulder was abducted to 90°. A five-point scale is used: 0 = no contraction of the biceps brachii; 1 = unable to reach the level of the nipples; 2 = unable to touch the mouth; 3 = able to repeat the task with severe or moderate clumsiness; 4 = able to repeat the task with mild clumsiness; 5 = able to repeat the task as smoothly as on the unaffected side.

3. Modified Ashworth Scale (MAS) [25]

This is a widely used scale for the assessment of the spasticity. Scoring is based on the resistance to passive stretch throughout the range of motion (ROM) of a joint (0 = no increase; 1 = slight increase at the end of the ROM; 1+ = slight increase throughout less than half of ROM; 2 = increase through most of ROM but easily moved; 3 = increase, passive movement difficult; 4 = rigid). In this study, we evaluated MAS in the elbow flexor muscles in the affected arm. We treated 1+ as 2 in the analysis of this study. Therefore, the score for analyses ranges from 0 to 5.

4. Wolf Motor Function Test (WMFT) [7]

This is a performance-based test that can evaluate upper extremity function through timed single- or multiple-joint motions and functional tasks with high reliability and validity [7, 26]. Each task item is timed and the amount of the time required to perform the tasks can be used as a quantitative measure of the WMFT (WMFT-time). Each item is also rated by the six-point Functional Ability Scale (FAS) (0 = no use; 1 = an attempt is made to use the arm; 2 = the arm does participate, but requires assistance of an uninvolved extremity; 3 = movement is performed slowly and/or with effort; 4 = movement is close to normal but slightly slower or may lack precision; 5 = normal) and the total score is considered as a qualitative score of the WMFT (WMFT-FAS). We included seven out of the fifteen motion tasks that require only the proximal function—not the finger function—for the analyses of this study: (1) forearm to table (side); (2) forearm to box (side); (3) extend elbow (to the side); (4) extend elbow (to the side, with weight); (5) hand to table (front); (6) hand to box (front); and (7) reach and retrieve (front). The FAS score ranges from 0 (worst) to 35 (best).

### Analysis

#### Differences and correlation of robotic measures between the paretic arm and the non-paretic arm

To explore robotic measures which have the potential to distinguish the paretic arm from the non-paretic arm, VGR parameters in the paretic arm were compared with those in the non-paretic arm with the Wilcoxon signed rank test. The effect size (ES) of the Wilcoxon rank sum test was calculated by dividing the Z-score by the square root of the total number of participants: ES = Z/sqrt(N). Then, to explore which robotic measures are related to motor control regardless of paresis, correlations between VGR parameters in the paretic arm and in the non-paretic arm were also examined with Spearman’s correlation coefficients.

#### Correlations between robotic measures and clinical assessments

To investigate the relationship between robotic measures and clinical assessments, correlations between the VGR parameters and clinical scales were investigated with Spearman’s rank correlation coefficients.

All statistical analyses were performed using STATA/SE 13.1 (StataCorp., Texas, USA). The strength of the correlation coefficients was interpreted according to Guilford [27]: 0.0–0.2 little if any; 0.2–0.4 weak; 0.4–0.7 moderate; 0.7–1.0 strong. Any p-values less than 0.05 were considered statistically significant.

## Results

### Differences and correlation of robotic measures between the paretic arm and the non-paretic arm

As shown in Table 3, eleven of the twelve parameters obtained from the VGR task in the paretic arm were statistically different from those in the non-paretic arm with large effect sizes (|ES| = 0.76–0.87), with the exception of Max Speed (ES = −0.42). Table 4 shows that 2 parameters (Reaction Time and Max Speed) had nearly moderate correlations with statistical significance between the paretic arm and the non-paretic arm.

**Table 3:** Comparison between parameters of Visually Guided Reaching task in paretic and non-paretic arms

Parameters	Paretic arm, median (range)	Non-paretic arm, median (range)	Wilcoxon signed rank test	
			*p*-values	Effect size
Posture Speed [×10^−2^ m/s]	0.58 (0.13−1.37)	0.35 (0.16−0.61)	<0.001	0.79
Reaction Time [s]	0.47 (0.32−0.67)	0.33 (0.25−0.42)	<0.001	0.87
No Reaction Time	5 (0–60)	0 (0–13)	<0.001	0.76
Initial Direction Error [×10^−2^ rad]	25.07 (3.71−94.67)	4.70 (2.20−10.43)	<0.001	0.87
Initial Distance Ratio	0.43 (0.15−0.89)	0.89 (0.54−1)	<0.001	−0.87
Initial Speed Ratio	0.84 (0.59−1)	1 (0.93−1)	<0.001	−0.85
Speed Maxima Count	4.36 (2.16−10)	2.38 (1.63−3.63)	<0.001	0.85
Min Max Speed [×10^−2^ m/s]	4.50 (0.84−19.27)	1.37 (0.52−4.33)	<0.001	0.86
Movement Time [s]	2.10 (1.02−3.67)	1.15 (0.82−1.62)	<0.001	0.87
Path Length Ratio	1.83 (1.11−5.95)	1.12 (1.05−1.42)	<0.001	0.86
Max Speed [×10^−2^ m/s]	19.29 (7.81−37.51)	22.45 (12.52−38.50)	0.003	−0.42
No Movement End	15 (0–63)	0 (0–13)	<0.001	0.78

*n* = 49. The data of participants who failed to reach the target during the trial (No Movement End = 64) (*n* = 6) or lacked any of the twelve parameters (*n* = 1) were excluded from data analysis

**Table 4:** Spearman’s rank correlation coefficients between parameters of Visually Guided Reaching in paretic and non-paretic arms

Parameters	Correlation coefficients
Posture Speed	0.24
Reaction Time	0.41^a^
No Reaction Time	0.10
Initial Direction Error	0.20
Initial Distance Ratio	0.03
Initial Speed Ratio	0.07
Speed Maxima Count	0.09
Min Max Speed	0.24
Movement Time	0.21
Path Length Ratio	0.03
Max Speed	0.42^a^
No Movement End	0.00

^a^ *p* < 0.01. *n* = 49

The data of participants who failed to reach the target during the trial (No Movement End = 64) (*n* = 6) or lacked any of the twelve parameters (*n* = 1) were excluded from data analysis

### Correlations between robotic measures and clinical assessments

**Fig. 1:**
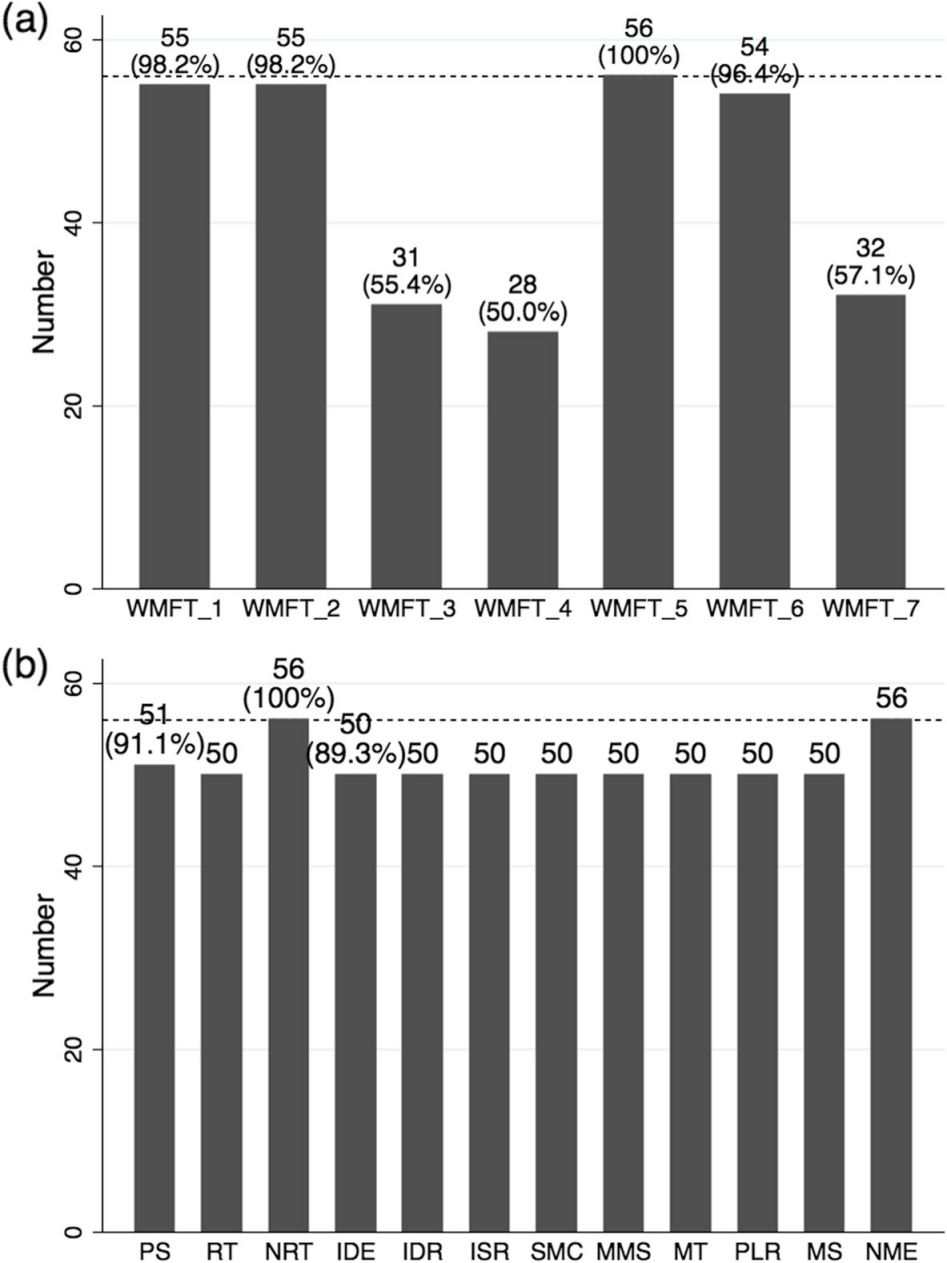
Number of participants with qualitative data from Wolf Motor Function Test and KINARM Exoskeleton. **a** The number of participants who could perform each task without any assistance in the Wolf Motor Function Test (i.e., score of the Functional Ability Scale = 3, 4, or 5). **b** The number of participants for whom each parameter in the Visually Guided Reaching task could be obtained. The maximum number of participants (56 participants = 100 %) are shown by the dotted line in each figure. PS, Posture Speed; RT, Reaction Time; NRT, No Reaction Time; IDE, Initial Direction Error; IDR, Initial Distance Ratio; ISR, Initial Speed Ratio; SMC, Speed Maxima Count; MMS, Min Max Speed; MT, Movement Time; PLR, Path Length Ratio; MS, Max Speed; NEM, No Movement End

As for WMFT, only 44.6 % (25 out of 56 participants) could complete all seven tasks without any assistance. Because performance time of a task assisted by the non-paretic arm or by therapists (FAS score ≤ 2) could not be treated as an exact quantitative evaluation, total performance time of all seven tasks was not considered to be suitable for analyses in this study. Instead, we adopted the sum of performance time in four out of the seven tasks (tasks 1, 2, 5, and 6) as WMFT-time, excluding tasks 3, 4, and 7, with which about half of the participants needed some assistance (see Fig. 1a). On the other hand, we could obtain all of the twelve parameters in the VGR task from 87.5 % of the participants (49 out of 56 participants). Each parameter could be obtained for nearly 90 % of participants (details are shown in Fig. 1b). Six participants failed to reach the target throughout the trial (No Movement End = 64) and one participant could not get nine out of twelve parameters.

Table 5 shows the correlations among the clinical scales. Correlations between clinical scales were generally moderate except for a strong correlation between FMA-UE(A) and WMFT-FAS (*r* = 0.72, *p* < 0.01). The MAS score had relatively weak correlation with the other scales and is thus considered to be focusing on somewhat different aspects, namely muscle tone, from what the others can describe (impairment and/or functional performance).

**Table 5:** Spearman’s rank correlation coefficients between clinical scales

	FMA-UE(A)	SIAS-KM	MAS elbow	WMFT -FAS (prox)	WMFT-time (1, 2, 5, 6) ^c^
FMA-UE(A)	1.00				
SIAS-KM	0.57^a^	1.00			
MAS elbow	−0.53^a^	−0.33^b^	1.00		
WMFT-FAS (prox)	0.72^a^	0.66^a^	−0.45^a^	1.00	
WMFT-time (1, 2, 5, 6) ^c^	−0.48^a^	−0.56^a^	0.29^b^	−0.58^a^	1.00

^a^ *p* < 0.01, ^b^ *p* < 0.05. ^c^The data of participants who required any assistance in performing tasks 1, 2, 5, or 6 were excluded (*n* = 2)

*FMA-UE(A)*, Part A of the Fugl-Meyer Assessment Upper Extremity; *SIAS-KM*, Knee-Mouth test of Stroke Impairment Assessment Set; *MAS*, Modified Ashworth Scale; *WMFT-FAS (prox)*, total score of Functional Ability Scale in tasks 1–7 of the Wolf Motor Function Test; *WMFT-time (1, 2, 5, 6)*, total performance time in tasks 1, 2, 5, and 6 of the Wolf Motor Function Test

Table 6 summarizes correlations between VGR parameters and clinical scales. FMA-UE(A) showed significant correlation with ten of the twelve VGR parameters, among which nine correlation coefficients were moderate (|r| = 0.40–0.60). The WMFT-FAS (|r| = 0.52–0.64) and SIAS-KM (|r| = 0.42–0.49) also showed significant and moderate correlations with eight of the twelve VGR parameters. MAS in the elbow correlated significantly with eight parameters and among them six were moderate (|r| = 0.44–0.48). The WMFT-time (tasks 1, 2, 5, and 6) was significantly but relatively weakly correlated with eight parameters (|r| = 0.34–0.47).

**Table 6:** Spearman’s rank correlation coefficients between clinical scales and parameters of the Visually Guided Reaching task

Parameters of VGR	FMA-UE(A)	SIAS-KM	MAS elbow	WMFT -FAS (prox)	WMFT -time (1, 2, 5, 6)
Posture Speed	−0.24	−0.04	0.03	−0.01	0.01
Reaction Time	−0.32^b^	−0.28	0.15	−0.20	0.19
No Reaction Time	−0.54 ^a^	−0.46 ^a^	0.27	−0.52 ^a^	0.37^b^
Initial Direction Error	−0.60 ^a^	−0.42 ^a^	0.46 ^a^	−0.57 ^a^	0.42 ^a^
Initial Distance Ratio	0.58 ^a^	0.43 ^a^	−0.47 ^a^	0.64 ^a^	−0.47 ^a^
Initial Speed Ratio	0.57 ^a^	0.42 ^a^	−0.47 ^a^	0.62 ^a^	−0.44 ^a^
Speed Maxima Count	−0.58 ^a^	−0.45 ^a^	0.47 ^a^	−0.59 ^a^	0.40 ^a^
Min Max Speed	−0.40 ^a^	−0.28	0.36^b^	−0.24	0.08
Movement Time	−0.52 ^a^	−0.45 ^a^	0.44 ^a^	−0.60 ^a^	0.40 ^a^
Path Length Ratio	−0.54 ^a^	−0.49 ^a^	0.48 ^a^	−0.53 ^a^	0.34^b^
Max Speed	−0.06	−0.09	−0.08	0.08	−0.14
No Movement End	−0.58 ^a^	−0.49 ^a^	0.37^b^	−0.58 ^a^	0.40 ^a^

^a^ *p* < 0.01, ^b^ *p* < 0.05

Underlined numbers are correlation coefficients higher than 0.40. *n* = 47. The data for participants who failed to reach the target during the trial (No Movement End = 64) or lacked any of the twelve parameters (*n* = 7) or who required any assistance in performing tasks 1, 2, 5, or 6 (*n* = 2) were excluded from data analysis

*FMA-UE(A)* Part A of the Fugl-Meyer Assessment Upper Extremity, *SIAS-KM* Knee-Mouth test of Stroke Impairment Assessment Set, *MAS* Modified Ashworth Scale; *WMFT-FAS (prox)* total score of Functional Ability Scale in task 1–7 of the Wolf Motor Function Test, *WMFT-time (1, 2, 5, 6)* total performance time in tasks 1, 2, 5, and 6 of the Wolf Motor Function Test

## Discussion

In this study, we examined the validity and clinical usefulness of reaching tasks in the KINARM Exoskeleton. Our findings indicate that the obtained measures have not only concurrent validity against common clinical scales, but also the possibility of providing more useful evaluations in severe hemiparetic patients than using conventional clinical scales.

To begin with, the comparison between the paretic and non-paretic arms revealed that robotic parameters could detect the presence of paresis very clearly in the assessed population, which is in accordance with the results described by Coderre et al. [14]. As a new point, we sought common features in the parameters of both sides by examining correlations between them. Interestingly, the values for the paretic arm and non-paretic arm had correlations in two parameters (Reaction Time and Max Speed), indicating that these parameters may detect factors other than paresis that affect motor control of bilateral sides in post-stroke patients.

With regard to the validity of robotic measures, we found significant correlations between the VGR parameters and clinically well-established scales that describe the severity of paresis, the FMA and the SIAS. The validity of the robotic measures derived from the KINARM Exoskeleton in terms of discriminability of abnormal from normal in patients with stroke has already been reported [14, 16]. However, the correlations between these robotic measures and the clinical scales for assessing impairments have not been well explored. In this study, we showed significant and moderate correlations between most of the robotic parameters and the clinical measures (9 with the FMA, 8 with the SIAS), and thus confirmed for the first time the concurrent validity of using the KINARM Exoskeleton for the assessment of the severity of paresis.

In addition, we found significant and moderate correlations between the VGR parameters and a part of the WMFT, which is a well-established clinical scale for assessing arm function. As introduced in the Background section, it has been reported that two clinical measures (the PPB and the FIM) correlated with the robotic measures [16]. However, correlations could be drawn by other confounding factors such as the severity of whole brain damage, because these measures do not directly reflect arm functions. Our results could ascertain for the first time the validity of the VGR parameters with clinical assessments focused on arm functions.

Among the twelve parameters of the VGR task, correlation coefficients with clinical assessments higher than 0.6 were seen in the indices of feed-forward control (Initial Direction Error, Initial Distance Ratio, and Initial Speed Ratio) and Movement Time as total movement metrics. As a parameter for this initial movement phase, Zollo et al. [28] proposed the aiming angle, defined as the angular difference between the initial target direction and the direction of travel in the task with the InMotion2 Robot. They found that aiming angle had a significant correlation with the FMA score. Our study revealed that more aspects of feed-forward control are impaired in hemiparetic arm movement— error in direction, inadequate distance to the target, and failure to attain peak velocity during the initial phase of movement. As for Movement Time, several other robotic devices adopt this type of measure [29–31]. In the KINARM Exoskeleton, this parameter can be used as a clinically validated continuous scale because our results were almost the same as those reported by Dukelow et al. [16].

Our findings are also in agreement with Colombo et al. [32] in that the number of peaks in the hand velocity profile in the MEMOS robot, similar to the Speed Maxima Count in the KINARM Exoskeleton, is correlated significantly with several clinical measures. Therefore, it might be a clinically useful scale regardless of the type of robotic device. Those multiple speed peaks within one reaching movement are caused by a sequence of small movements. Krebs et al. [33] and Rohrer et al. [34] termed those small movements ‘submovements,’ and a decrease in submovements, which means improvement in smoothness, can be considered as the result of a learned process rather than a natural consequence of the neuromuscular system. Interestingly, this parameter has been understood in two different contexts: as a parameter of feedback control as in the KINARM Exoskeleton and as a parameter of movement smoothness, as in the MEMOS robot. Further studies are needed to explore the meaning of this new clinical measure in practice and to investigate the underlying mechanisms of the submovements.

As for the clinical usefulness of the robotic measures compared to the conventional clinical scales, we point out two important facts. Firstly, the correlation coefficients between the eight VGR parameters and the FMA-UE(A) (|r| = 0.52−0.60) were stronger than those between the WMFT-time (tasks 1, 2, 5, and 6) and the FMA-UE(A) (|r| = 0.48), which means that the VGR parameters were more sensitive in reflecting the FMA scores than the WMFT tasks (tasks 1, 2, 5, and 6). Secondly, as shown in Fig. 1, we could obtain more quantitative data from robotic tasks than from the WMFT. Hence it is suggested that this robotic task may have the potential to assess motor impairment quantitatively with better accuracy than the WMFT, which is one of the conventional clinical quantitative scales in use. More importantly, these facts allow rehabilitation practitioners to make use of the measurements provided by this type of a robot to quantify upper extremity functions in post-stroke patients with severe hemiparesis who are difficult to assess in detail with existing clinical tasks.

The results of this study show that the measures obtained from the two-dimensional reaching task with reduced degrees of freedom in the KINARM Exoskeleton and the clinical scales in three dimensions have significant correlations. It means that compensating for the effect of gravity by supporting the limb and decreasing the degrees of freedom of the joints allows clinically relevant evaluation of hemiparetic patients. On the other hand, this weight-supporting system may potentially fail to detect certain aspects of impairment in the paretic arm. Relatively low correlations between the robotic measures and the SIAS-KM, which is examined by a single anti-gravitational task, might support this hypothesis.

In this study, we investigated chronic post-stroke patients; this differs from previous reports, which assessed patients in the subacute phase. In addition, the MAS scores of our participants were relatively higher compared to those of the participants in Dukelow’s study [16]. As shown in Table 1, nearly 50 % of our participants marked MAS scores of 1+ or 2. On the contrary, the MAS scores in around 80 % of their participants were rated as 0 or 1 [16]. Despite these differences, the robotic measures have significant correlations with clinical scales assessing the severity of paresis or reaching function of the arm. The results indicate that the KINARM Exoskeleton can be used practically, even in the chronic phase after a stroke with muscle spasticity.

On the other hand, correlation between the robotic measures and the MAS score in the affected elbow flexor muscles itself was relatively low. The MAS score showed moderate correlation with some parameters of feed-forward and feedback control, but not with two of the measures (No Reaction Time and No Movement End) which correlated more strongly with the other clinical scales. Similarly, Bosecker et al. reported that the MAS scores were poorly estimated by the linear regression model developed from kinematic and kinetic measures of the InMotion2 robot [29]. Therefore, the MAS score, which represents muscle spasticity, may have an effect on reaching movement in a different way from that in which paresis affects motor control. Alternatively, poor correlations between the robotic measures and the MAS score may be due to the weight-supported conditions of the robotic device, as clinicians often observe that muscle tone is reduced in weight-supported conditions. Further study is required to reveal the effect of muscle spasticity on motor control in post-stroke patients.

This study has several limitations. Firstly, all participants enrolled in the study suffered from chronic stroke; therefore, generalization of our results to acute or subacute patients is unknown. Secondly, we had no control participants to compare with the hemiparetic patients. Another study design will be required to compare the detectability of slight impairment between robotic and conventional assessments. Thirdly, we did not obtain the clinical scales from the non-paretic arm. Investigation including the correlations between the clinical scores and the VGR parameters in the non-paretic arms would add more insight into the usability of robotic assessments. Finally, the design of this study is cross-sectional. A longitudinal design is needed to compare responsiveness between robotic and conventional assessments.

## Conclusions

This study showed the clinical usefulness and validity of robotic measures obtained from a reaching task in the KINARM Exoskeleton. These measures not only provided objective, quantitative, and continuous assessments of hemiparetic arm movement, but also had stronger correlation with measures of motor impairment than existing quantitative scales of functional ability. Our findings indicate that these robotic assessments through two-dimensional tasks in the weight-supported condition can provide clinically meaningful evaluations as well as information about the mechanism of reaching movement motor control in post-stroke patients. This is an important step toward bringing robotic technologies into the clinical rehabilitation setting.
